# Prevalence of Chagas disease in Colombia: A systematic review and meta-analysis

**DOI:** 10.1371/journal.pone.0210156

**Published:** 2019-01-07

**Authors:** Mario J. Olivera, Johana A. Fory, Julián F. Porras, Giancarlo Buitrago

**Affiliations:** 1 Grupo de Parasitología, Instituto Nacional de Salud, Bogotá, D.C., Colombia; 2 Programme in Health Economics, Pontificia Universidad Javeriana, Bogotá, D.C., Colombia; 3 Facultad de Medicina, Universidad Militar Nueva Granada, Bogotá, D.C., Colombia; 4 Facultad de Medicina, Universidad Nacional de Colombia, Bogotá, D.C., Colombia; 5 Departamento de Epidemiología y Bioestadística, Pontificia Universidad Javeriana, Bogotá, D.C., Colombia; Universite de Perpignan, FRANCE

## Abstract

**Background:**

Despite the adoption of campaigns to interrupt the main vector and to detect *Trypanosoma cruzi* in blood banks, millions of people are still chronically infected; however, the prevalence data are limited, and the epidemiology of Chagas disease has not been systematically evaluated. This study aimed to estimate the prevalence of Chagas disease in Colombia.

**Methods:**

A systematic literature review and meta-analysis was conducted to select all observational studies reporting the prevalence of Chagas disease in Colombia, based on serological diagnosis in participants of any age and published between January 2007 and November 2017. Pooled estimates and 95% confidence intervals (95% CIs) were calculated using random-effects models. In addition, the I^2^ statistic was calculated.

**Results:**

The literature search yielded a total of 1,510 studies; sixteen articles with relevant prevalence data were included in the systematic review. Of these, only 12 articles were included for entry in the meta-analysis. The pooled prevalence of Chagas disease across studies was 2.0% (95% CI: 1.0–4.0). A high degree of heterogeneity was found among studies (I^2^ > 75%; p < 0.001). The publication bias was not statistically significant (Egger’s test, p = 0.078). The highest pooled prevalences were found in the adult population (3.0%, 95% CI: 1.0–4.0), pregnant women (3.0%, 95% CI: 3.0–4.0) and the Orinoco region (7.0%, 95% CI: 2.2–12.6).

**Conclusions:**

The results indicate that the *T*. *cruzi*-infected population is aging, the adult population, pregnant women and that the Orinoco region (department of Casanare) have the highest prevalences. These results highlight the need to maintain screening and surveillance programs to identify people with chronic *T*. *cruzi* infections.

## Introduction

Chagas disease, an infection caused by the parasite *Trypanosoma cruzi*, affects 6–7 million people worldwide, and the infection is estimated to cause more than 7,000 deaths annually [1]. Despite decades of control efforts, which have reduced its incidence, millions of people are chronically infected in the most productive stages of their lives, which leads to job losses and the perpetuation of the cycle of poverty [1,2].

American trypanosomiasis is endemic in Latin America, where it is transmitted to animals and humans by triatomine insects that are found only in the Americas [3]. However, Chagas disease has become an emerging global problem due to the increasing international migration and travel of Latin Americans to non-endemic countries, particularly to regions of North America and Europe [4].

Chagas heart disease contributes significantly to the global burden of cardiovascular disease and is one of the leading causes non-ischemic cardiomyopathy in Latin America [5]. A large proportion of the burden of Chagas disease can remain hidden for years; up to 70% of infected people can be asymptomatic for more than three decades [6]. Many of these people do not know that they are infected, and a serological diagnosis is required, a procedure that is not routine in most primary care centers [7,8].

Once there is dilated cardiomyopathy, the most important and most severe manifestation of human chronic Chagas disease, the risk of death and disability increases [6,9,10]. This condition is characterized by heart failure, ventricular arrhythmias, heart block, thromboembolic phenomena and sudden death [9]. This dilated cardiomyopathy generates large direct costs, the loss of productivity and poor health-related quality of life [11,12].

It is important for Colombia to know the prevalence of Chagas disease and, in that way, to prioritize the political strategies that increase the efforts of access to diagnosis and timely treatment among populations at risk. This study aimed to estimate the prevalence of Chagas disease in Colombia, through a systematic review and meta-analysis.

## Methods

The PRISMA guidelines were followed in conducting and reporting the results of this systematic review and meta-analysis (S1 File). There is no previously published review protocol.

### Eligibility criteria

In this review, the eligibility criteria included the following: i) Cross-sectional, case-control or cohort studies carried out in Colombia reporting the prevalence of Chagas disease in any age range and ii) diagnosis confirmed as positive for *T*. *cruzi* infection by two independent serological tests (ELISA serology and indirect immunofluorescence or indirect hemagglutination) following international recommendations [13].

Studies were excluded for the review by the following criteria: i) they were conducted among populations of Colombian origin residing outside of Colombia, ii) they were not performed with human participants, iii) the samples used originated from sources other than the general geographical population or iv) they were reviews.

### Information sources

The following electronic databases were included in the search of the published literature: PubMed (US National Library of Medicine, National Institutes of Health), EMBASE (Excerpta Medica dataBASE), LILACS (Latin American and Caribbean Health Sciences Literature), and Scielo (Scientific Electronic Library Online) using MeSH terms and Entrees for PubMed and Embase and DeCS for the other two databases. In addition, Google Scholar was used to complement the search results. S1 Table shows the full search strategy for each database.

### Search strategy

All searches were conducted independently by two authors, and there were no disagreements between the authors. The key words “Chagas disease”, “*Trypanosoma cruzi*” and “American trypanosomiasis” were used individually and combined with each of the following: “Prevalence”, “Epidemiology” and “Colombia”. A secondary search was conducted by reviewing the reference lists of the included articles to identify additional relevant studies. The publication year range was limited to January 2007 to November 2017. Moreover, the language of the articles was restricted to articles written in English or Spanish. In the search conducted in Google Scholar, the first hundred articles were screened.

### Study selection and data collection

Two authors separately identified the articles, sequentially screened their titles and abstracts for eligibility and read the full texts of potentially relevant studies. Disagreements were resolved by consensus.

Data were extracted independently by both authors and were entered in a pre-tested database (Access 2007, Microsoft Inc., Redmond, WA). For each paper, the following items were extracted: first author, country, year of publication, research design, sample size, year data collection started, year data collection ended, the characteristics of the scenarios and the participants of the studies (e.g., location, age, sex), denominator (number of cases at risk), numerator (number of cases with Chagas disease) and prevalence.

### Assessing bias

All studies were assessed for quality and bias in the data analysis using an adapted version of the Risk of Bias Tool for Prevalence Studies developed by Hoy et al [14]. The tool includes ten parameters that assess measurement bias, selection bias, and bias related to the analysis. Each parameter was assessed as either low or high risk of bias, and unclear was regarded as high risk of bias. The overall risk of bias was then scored according to the number of high risk of bias parameters per study: low (≤ 2), medium (3–4) and high (≥ 5) [15]. This tool has been demonstrated to have high inter-rater agreement in judging the risk of bias for each item [14].

### Statistical analysis

The prevalence of Chagas disease was calculated for each geographic region using the number of cases reported in the sample as the numerator, divided by the total sample size as the denominator. All rates were calculated as the rate of Chagas per 100 people, and the total sample is the sum of the cases with Chagas and cases without Chagas. A random-effects model was used to aggregate individual effect sizes to create a pooled prevalence of Chagas disease reported as percentages and their 95% confidence intervals (95% CI), as well as to estimate the prevalence of Chagas disease for the general population. A forest plot was constructed to illustrate the prevalence of each study as well as pooled effects. Heterogeneity among studies was quantitatively assessed using the χ^2^ test on Cochran’s Q (reported as 2 and p values) and I^2^ statistics, which describes the percentage of variation in prevalence attributable to between-study heterogeneity (an I^2^ value of >75% is interpreted as high heterogeneity). Univariate analyses were performed on all variables, those with a result of p < 0.2 were selected for inclusion in multivariate models. Sensitivity analyses using the meta-regression method were carried out on the variables: study region (Amazon, Andean, Caribbean and Orinoco), sex report of the participants (men and women), sample size (≤ 400), year of publication (2007–2013 and 2014–2017) and year of data collection (2003–2010 and 2011–2017) to explore potential sources of heterogeneity. Finally, potential publication bias was investigated by means of funnel plots and Egger’s test of asymmetry. Each point in the funnel plots represents a study, its effect measure, or the prevalence and standard error. A logit transformation of prevalence was performed for each included study to analyze the publication bias. All the analyses were performed using the statistical package Stata, version 13.0 (Stata Corp LP, College Station, TX, USA). Thematic maps presenting the study sites and prevalence rate estimates for Colombian departments were created using the Geographic Resources Analysis Support System (GRASS) Software, version 9.3 (Open Source Geospatial Foundation).

## Results

### Selection of studies

**Fig 1:**
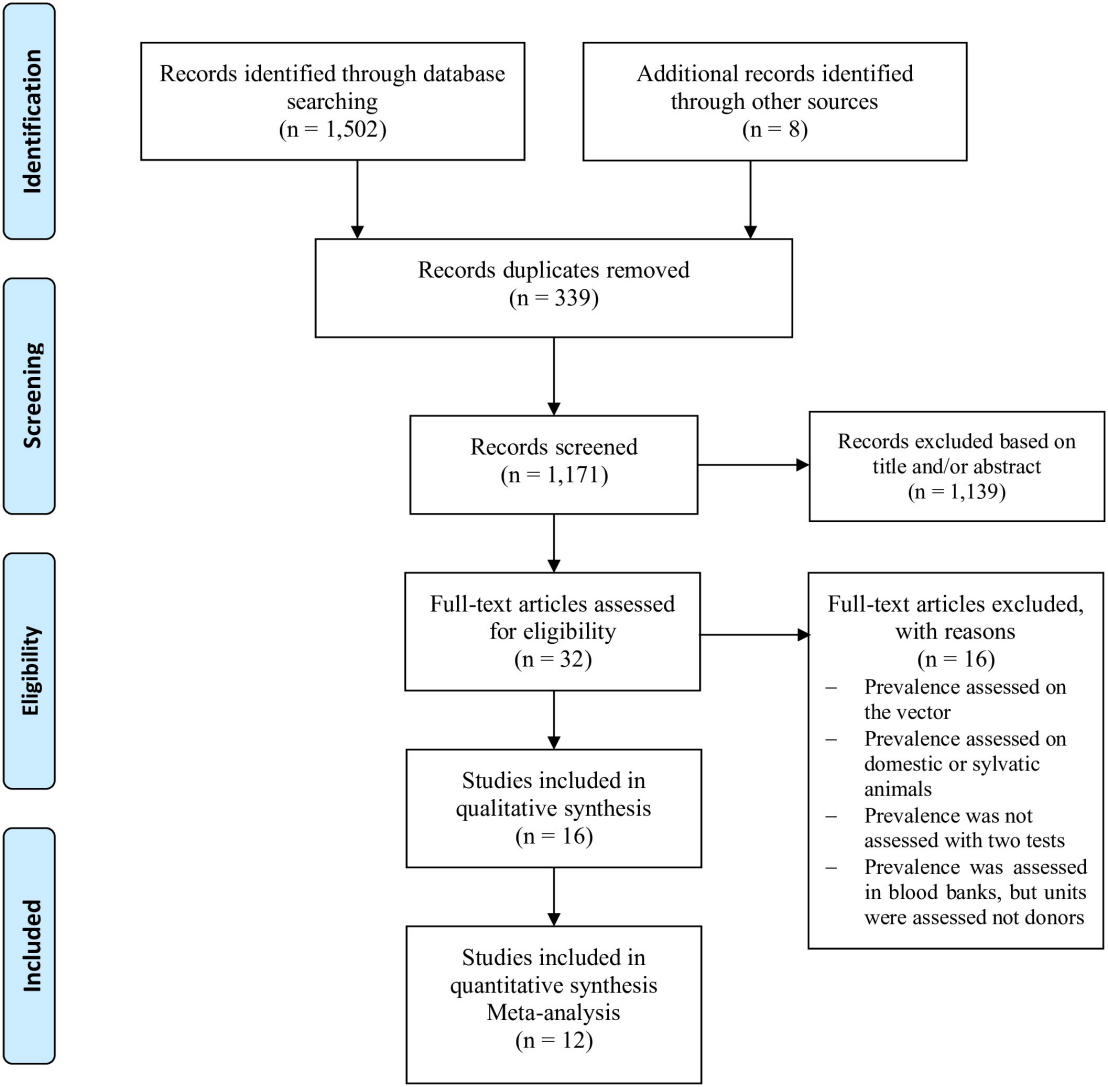
Flow diagram of the search and selection of studies on the prevalence of Chagas disease in Colombia from 2007 to November 2017.

The systematic literature search yielded 1,510 studies (Fig 1). After removing duplicates, 1,171 studies remained. On screening titles and abstracts for relevance, 1,139 studies were excluded, giving a total of 32 full texts that were assessed. A final set of sixteen studies were included in the systematic review. Of these, only 12 articles were included for entry in the meta-analysis.

### Study characteristics

**Fig 2:**
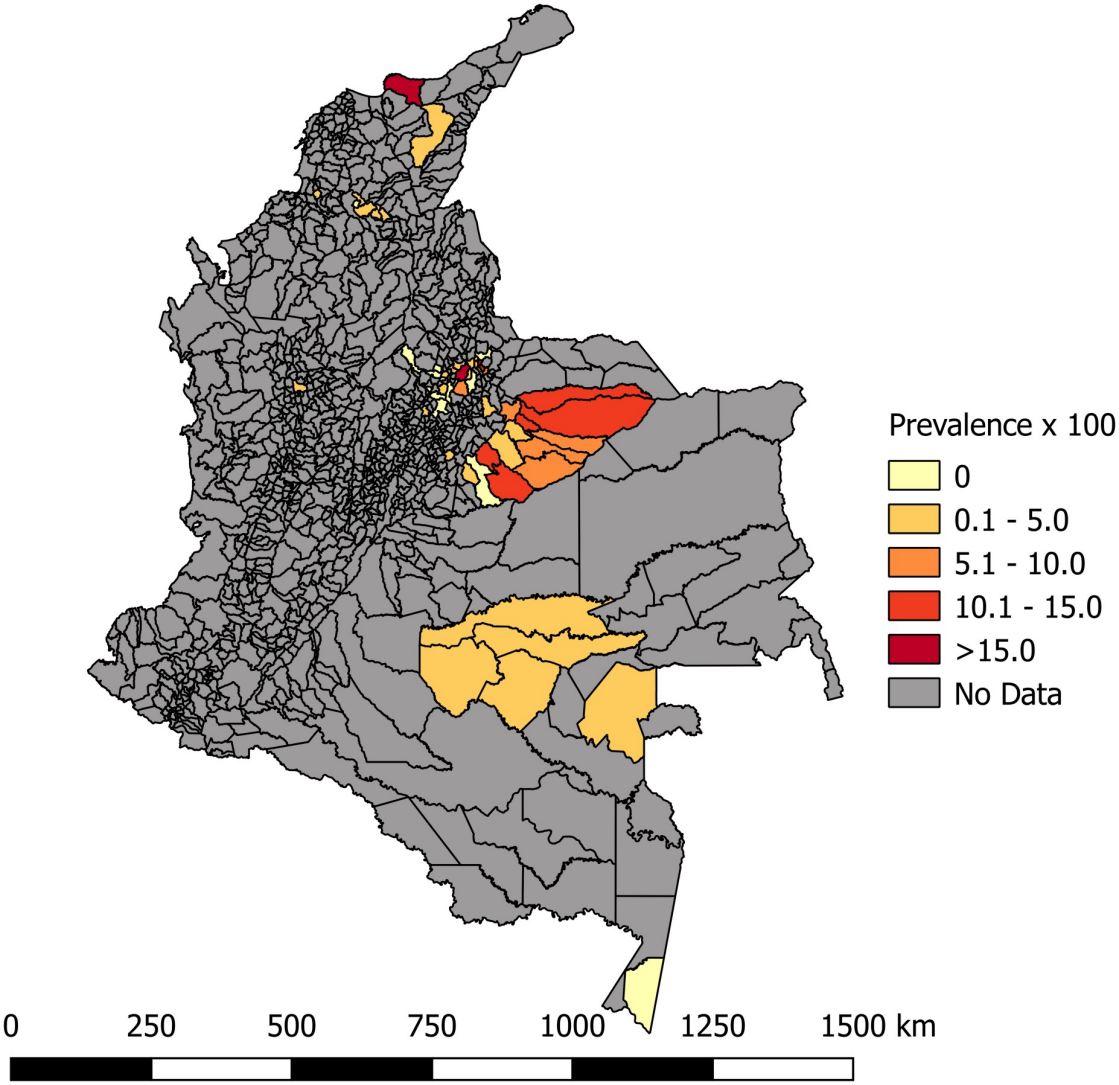
Map of Colombia showing the distribution of observed Chagas disease prevalence in studies published from 2007 to November 2017.

**Fig 3:**
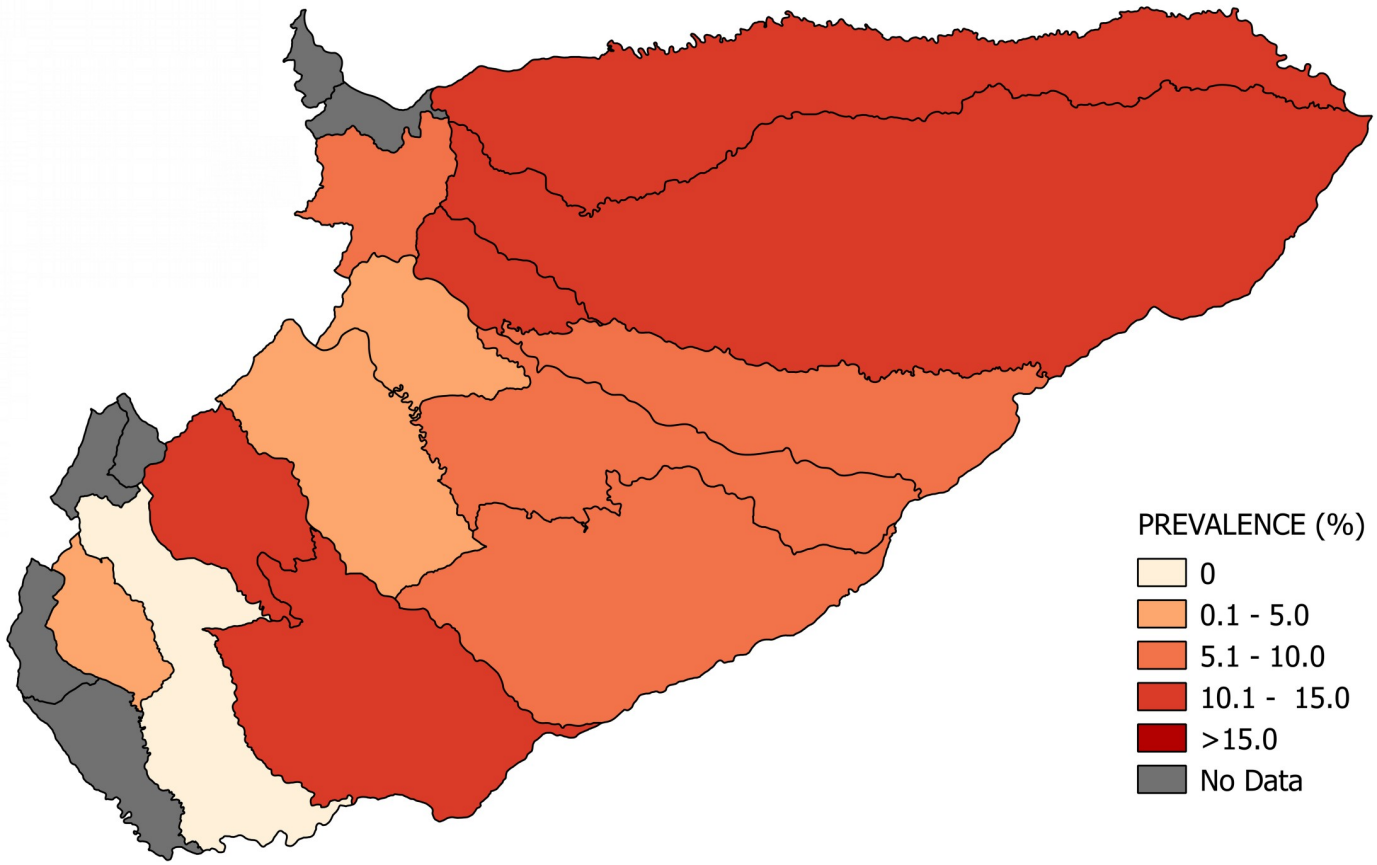
Map of the municipalities of Casanare showing the distribution of observed Chagas disease prevalence in studies published from 2007 to November 2017.

**Fig 4:**
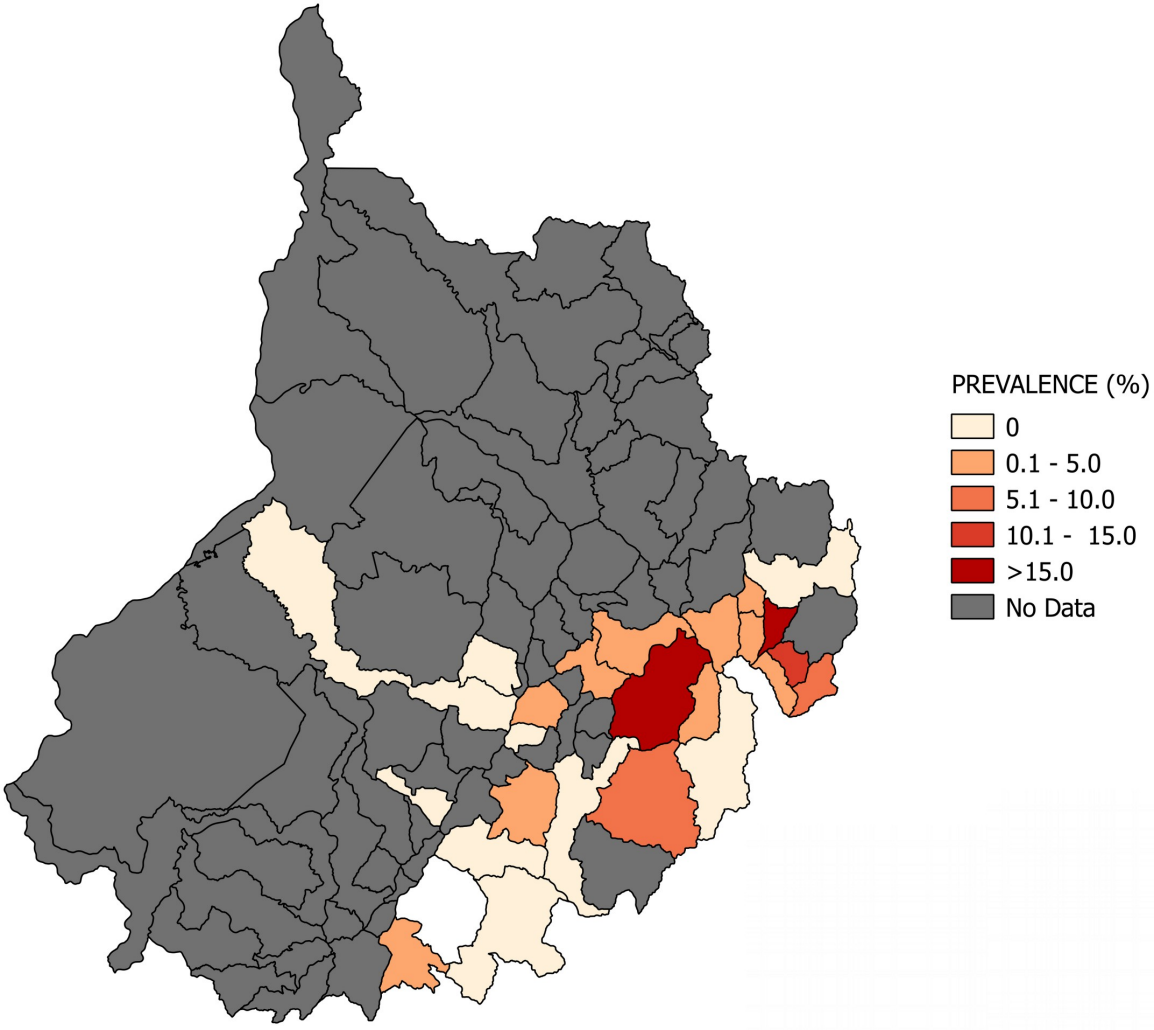
Map of the municipalities of Santander showing the distribution of observed Chagas disease prevalence in studies published from 2007 to November 2017.

**Fig 5:**
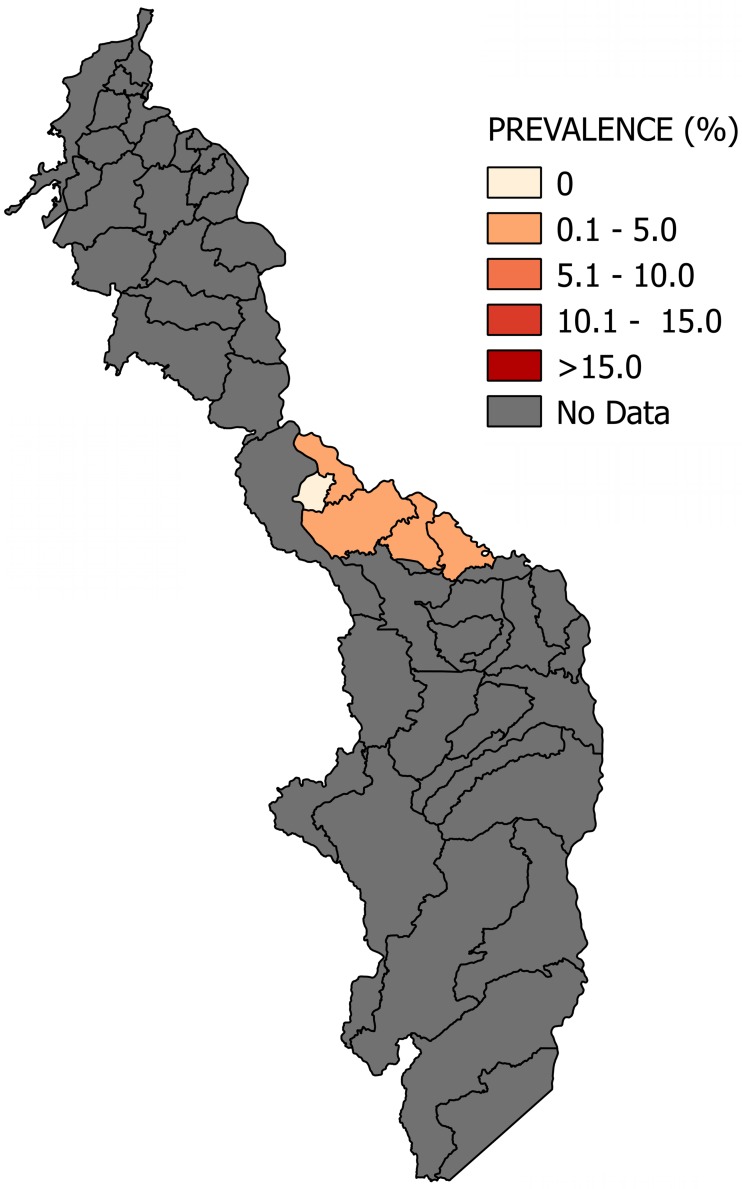
Map of the municipalities of Bolívar showing the distribution of observed Chagas disease prevalence in studies published from 2007 to November 2017.

**Fig 6:**
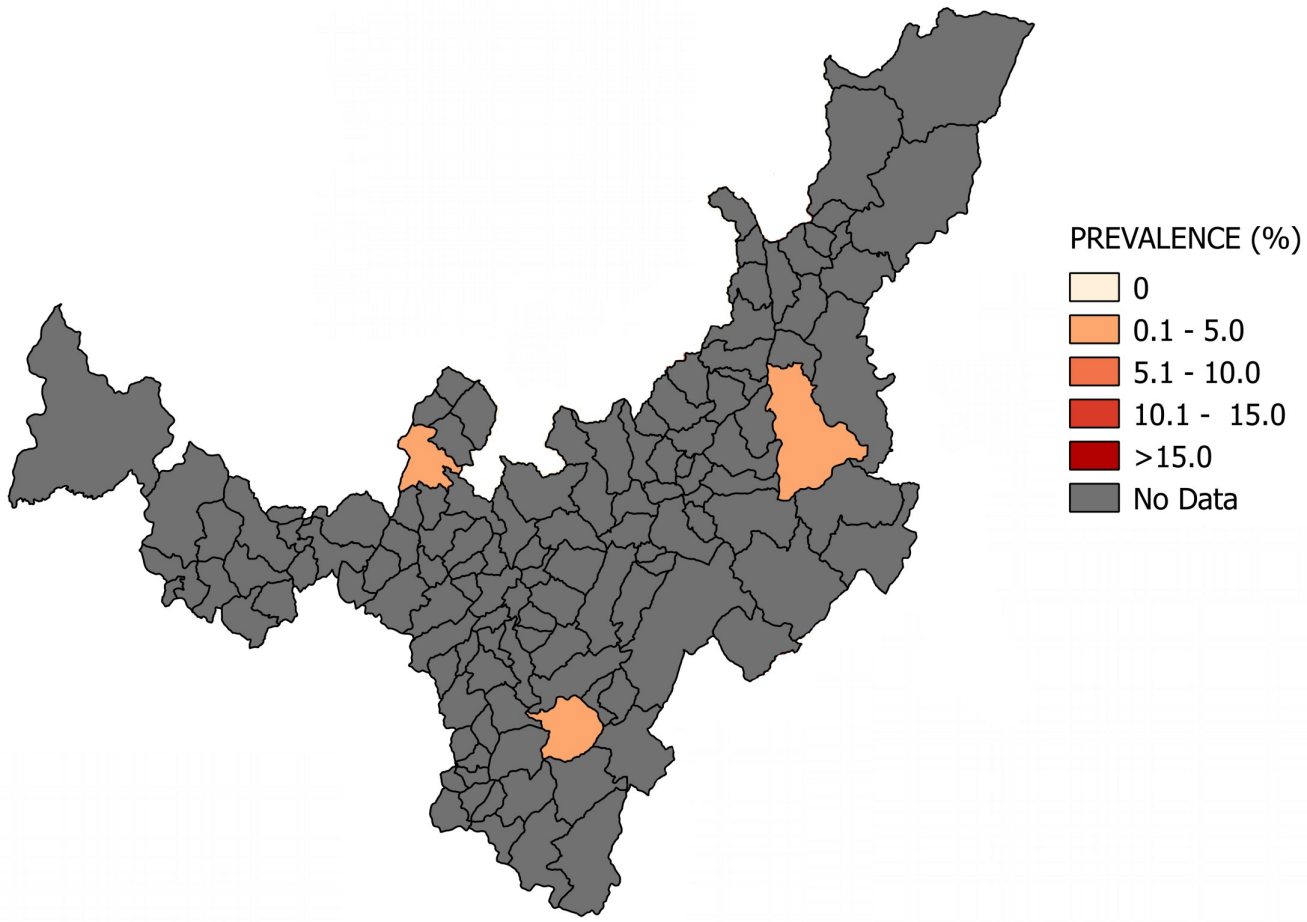
Map of the municipalities of Boyacá showing the distribution of observed Chagas disease prevalence in studies published from 2007 to November 2017.

**Fig 7:**
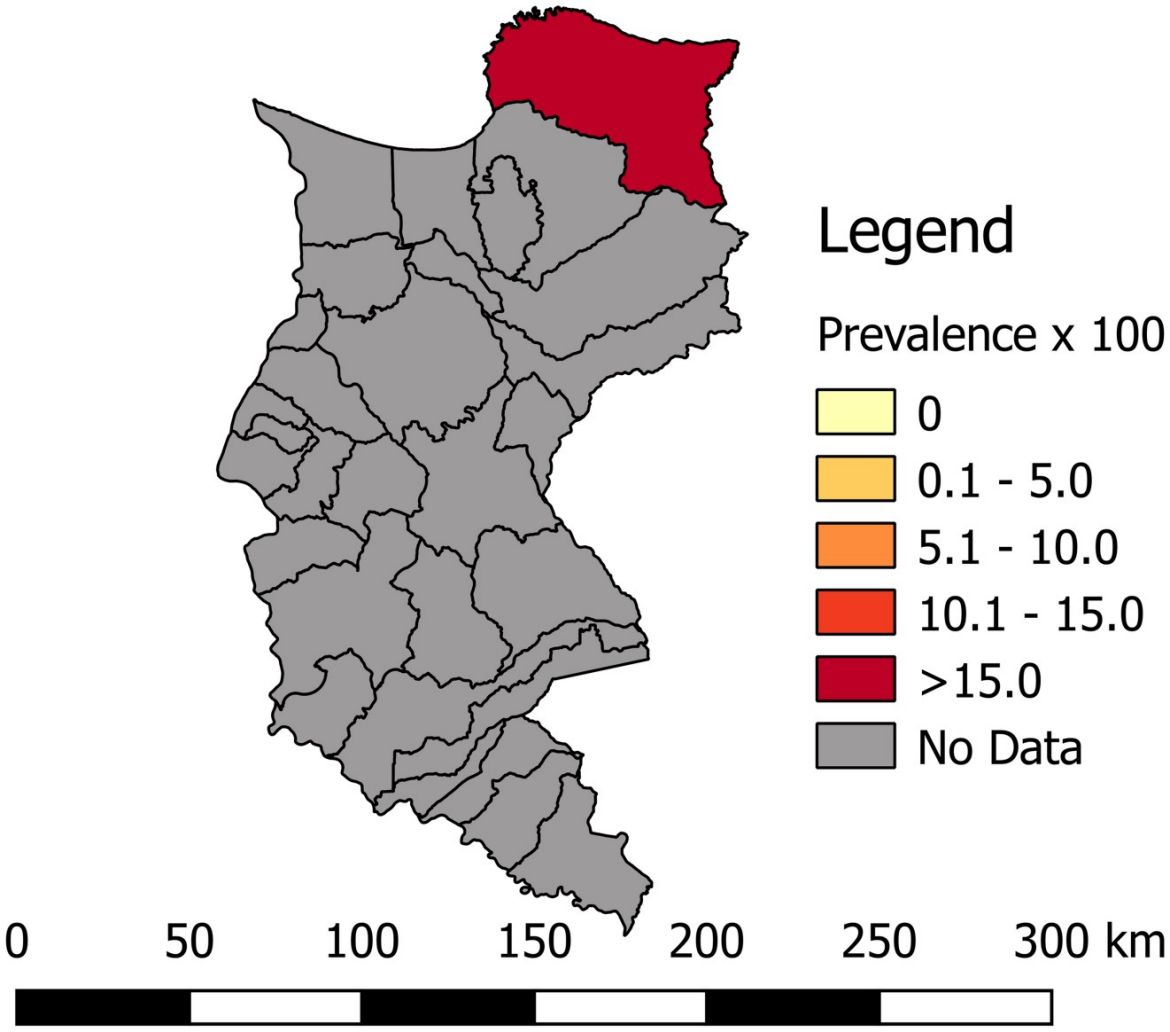
Map of the municipalities of Magdalena showing the distribution of observed Chagas disease prevalence in studies published from 2007 to November 2017.

**Fig 8:**
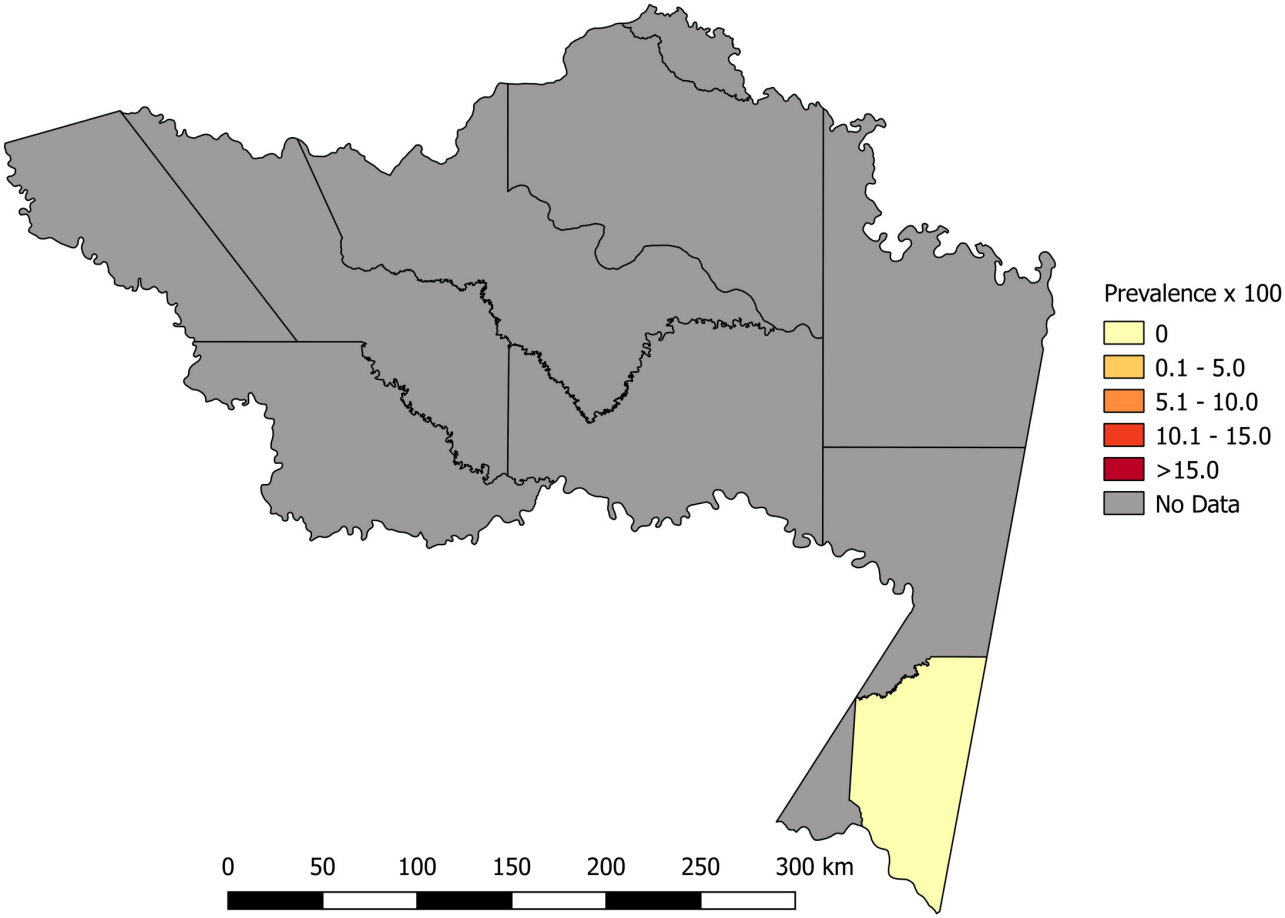
Map of the municipalities of Amazonas showing the distribution of observed Chagas disease prevalence in studies published from 2007 to November 2017.

Most of the studies were conducted across the main regions of Colombia (Amazon, Andean, Caribbean and Orinoco), predominantly in the Andean region (six studies). A total of 65 study sites were identified; 23 were in the department of Santander. The geographical distribution of the study sites and the observed prevalence by location are shown in Figs 2–8. Most of the included studies had cross-sectional designs, populations of both sexes, a broad age range in the adult population, and both urban and rural populations. The prevalence of Chagas disease varied from 0.0% to 36.9%. An overview of the included studies on the prevalence of Chagas disease is provided in Table 1 and S2 Table.

**Table 1:** Characteristics of Chagas disease prevalence studies from Colombia published from 2007 to November 2017.

Authors, publication year	Age (years)	Study site	Region	Sample size	Cases	Prevalence (%)
Suescún-Carrero et al. 2017 [16]	14–48	Boyacá	Andes	566	14	2.5
Flórez et al. 2016 [17]	2–64	Amazonas Guaviare Vaupés	Amazon	3429	34	0.9
Angulo-Silva et al. 2016 [18]	0–88	Casanare	Orinoco	492	57	11.6
Monroy et al. 2016 [19]	32±9.9	Boyacá	Andes	138	2	1.4
Castellanos-Domínguez et al. 2016 [20]	13–46	Santander	Andes	1,518	49	3.2
Bianchi et al. 2015 [21]	4–19	Casanare	Orinoco	3,033	62	2.0
Cantillo-Barraza et al. 2015 [22]	<15	Bolívar	Caribbean	803	2	0.2
Mejía-Jaramillo et al. 2014 [23]	NR	Sierra Nevada de Santa Marta	Caribbean	214	79	36.9
Cantillo-Barraza et al. 2014 [24]	1–91	Bolívar	Caribbean	743	13	1.7
Rocha-Muñoz et al. 2014 [25]	18–65	Cesar	Andes	16,661	24	0.1
Gutierrez et al. 2013 [26]	15–89	Casanare	Orinoco	486	75	15.4
Manrique-Abril et al. 2013 [27]	14–43	Boyacá	Andes	659	22	3.3
Bedoya et al. 2012 [28]	18–65	Antioquia	Andes	54,499	4	0.0
Cucunubá et al. 2012 [29]	13–46	Casanare	Orinoco	982	39	4.0
Rios-Osorio et al. 2012 [30]	NR	Sierra Nevada de Santa Marta	Caribbean	355	119	33.5
Hoyos et al. 2007 [31]	NR	Sucre	Caribbean	122	1	0.8

NR: Not reported

### Sensitivity and meta-regression analyses

The prevalence estimates were materially changed by excluding: four studies with sample size ≤400; eight studies that did not report sex of participants; and two study that did not report year of data collection (S3 Table). Significant heterogeneities remained (I^2^ = 99.2%). As summarized in S4 Table, except for sample size (p = 0.185) and Orinoco (p = 0.182), none of aforementioned variables were significantly associated with the detected heterogeneity in the univariate meta-regression (S1 Fig). Two variables, sample size and region (Orinoco), were then selected in the multivariate meta-regression; however, none of these variables were significantly associated with the detected heterogeneity.

### Risk of bias

**Fig 9:**
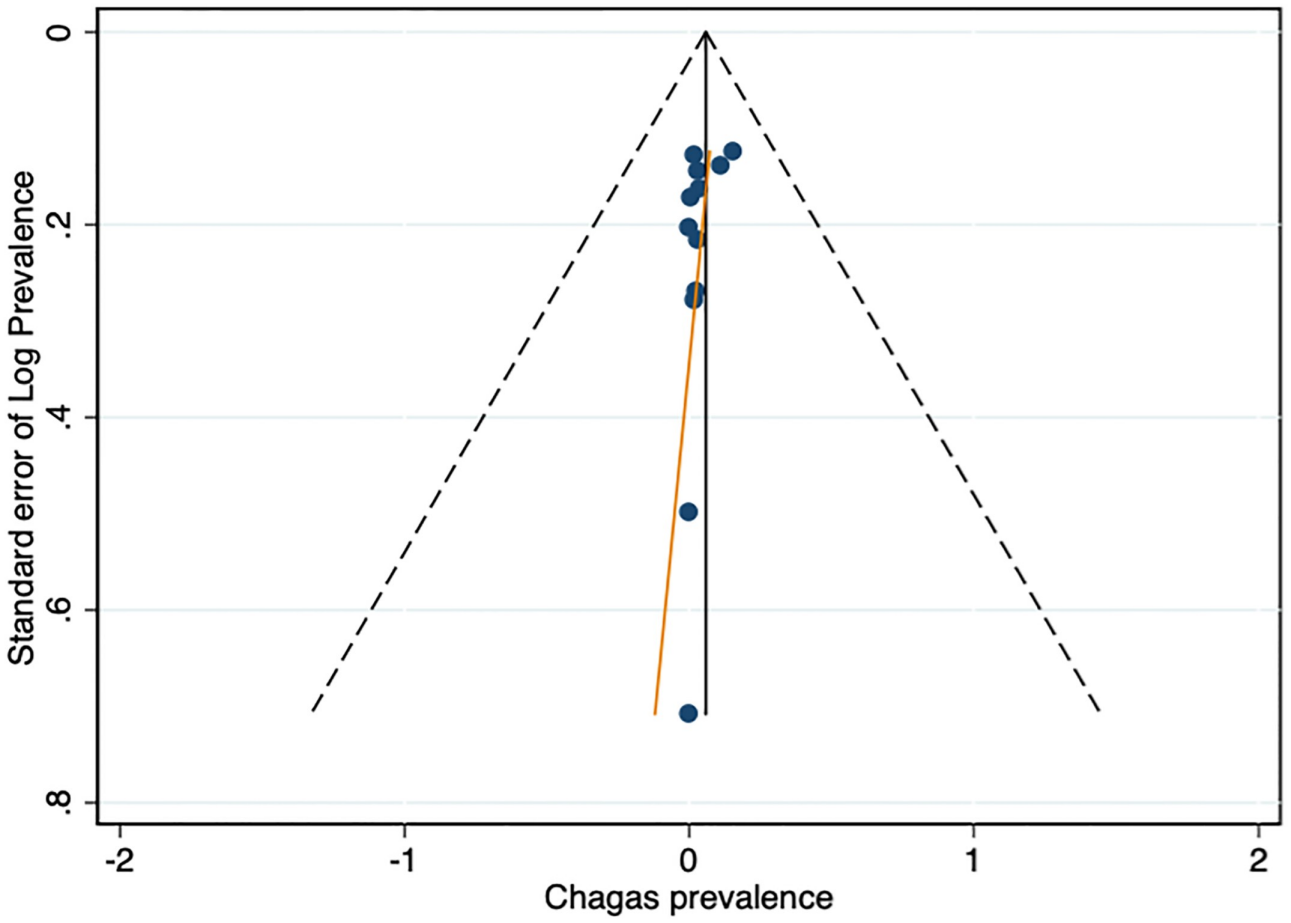
Funnel plot of 12 studies included in the meta-analysis.

Of the 12 included studies, five (41.7%) were rated as having a low risk of bias, three had a medium risk of bias (25.0%), and four had a high risk of bias (33.3%). High risk-of-bias ratings were most common for item 1 (national representativeness), item 2 (target population) and item 3 (random selection). Detailed results from the quality assessment of the twelve studies are presented in S5 Table. Due to the small number of articles identified, studies were not excluded from the primary analysis based on their quality assessment. The shape of funnel plot did not reveal obvious asymmetry (Fig 9). The results of Egger’s test also showed little evidence of significant publication bias among the contributing studies (p = 0.078).

### Pooled prevalence estimates

**Fig 10:**
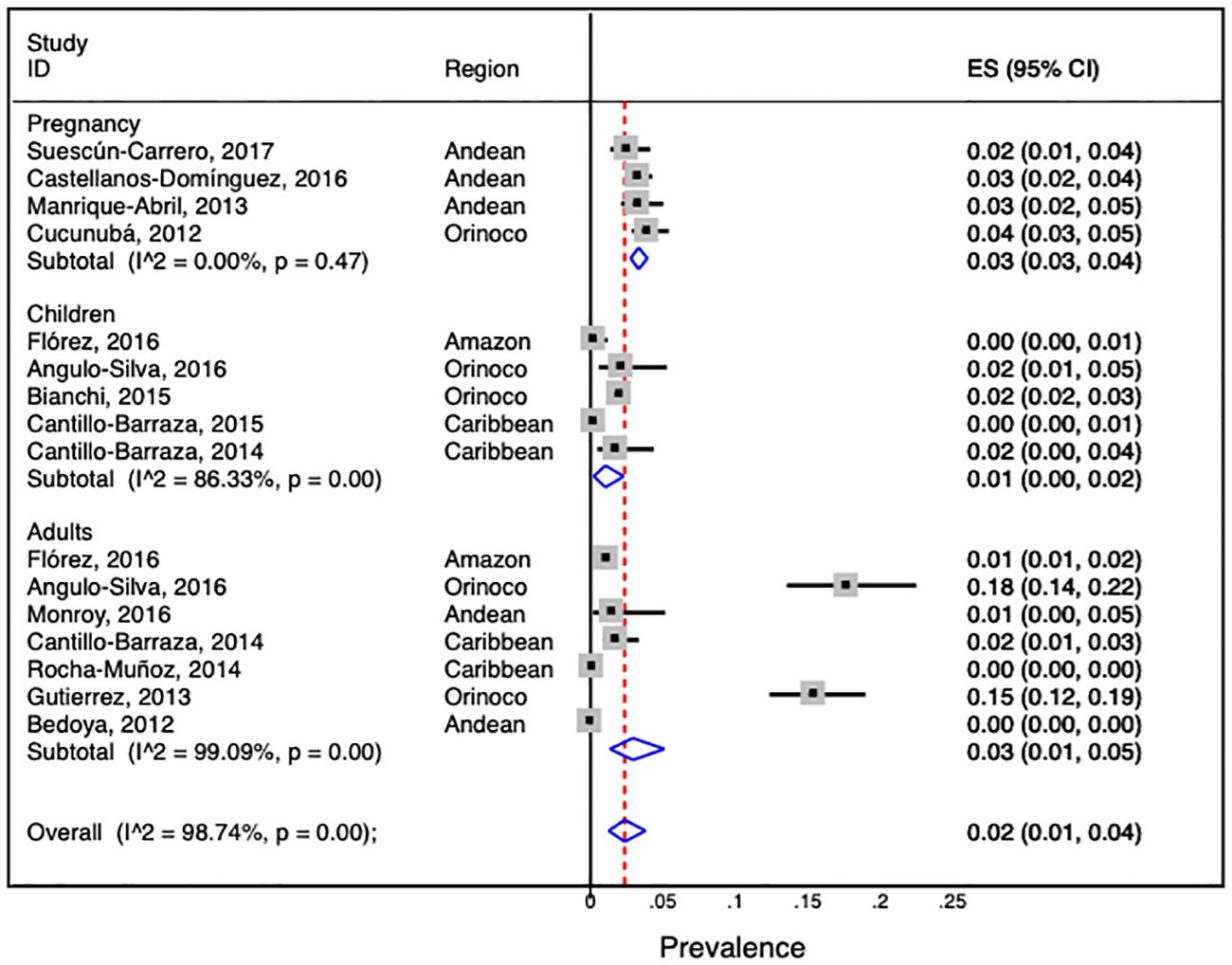
Forest plot showing the meta-analysis of population-based prevalence of Chagas disease in Colombia.

The overall prevalence of Chagas disease observed across the all studies was 2.0% (95% CI: 1.0–4.0). The observed I^2^ statistic showed a high and significant heterogeneity among studies (I^2^: 98.7%, p < 0.001), indicating great variability in effect size estimates (Fig 10). Sub-analysis by geographical region showed that the Orinoco had the highest prevalence (7.0%, 95% CI: 2.2–12.6; I^2^: 97.6%, p < 0.001). Not a single study was identified that included the Pacific region.

The subgroup analysis showed wide variations in prevalence between the clinical groups, concentrating the highest prevalence in the categories that included the adult population (3.0%, 95% CI: 1.0–5.0; I^2^: 99.0%, p <0.001) and pregnant women (3.0%, 95% CI: 3.0–4.0; I^2^: 0.0%, p 0.47) While lower prevalence was estimated in the category that included the children (1.0%, 95% CI: 0.0–2.0; I^2^: 86.3%, p <0.001). However, the lowest prevalence was estimated in the group of blood donors (0.5%, 95% CI: 0.0–1.4; I^2^: 99.4%, p <0.001).

These results also showed how the heterogeneity varied among the population groups, with the greatest heterogeneity evidenced in the categories of blood donors (I^2^: 99.4%, p < 0.001), the adult population (I^2^: 99.0%, p < 0.001) and the infant population (I^2^: 86.3%, p < 0.001). The lowest degree of heterogeneity was present in the pregnant women (I^2^: 0%, p = 0.47), which was not statistically significant.

According to the information available about sex distribution (eight studies), there was not enough evidence to support a difference between the prevalences of men and women. Compared to younger age-groups, adults (> 15-year-olds) had the highest prevalence of 3.0% (95% CI: 2.0–5.0; I^2^: 99.0%, p < 0.001), while the lowest prevalence, 1.0% (95% CI: 0.2–5.0; I^2^: 86.3%, p <0.001), was identified in the 0-15-year-olds.

## Discussion

This comprehensive systematic review with meta-analysis provides a comprehensive overview of the prevalence of Chagas disease in Colombia, over a period of more than a decade, by criteria of diagnosis of *T*. *cruzi* in chronic infections. The data indicate that the heterogeneity among the included studies was significant, given that the studies were conducted in different geographical regions, with heterogeneous population and different environmental exposure.

The pooled prevalence estimates for Chagas disease of 2.0% (95% CI: 1.0–4.0) was calculated based on 12 studies published between 2007 and 2017 that had from a broad range of research quality and sample sizes. Articles published before 2007 were not included in this review because the cases confirmed in the studies conducted before this date were not reported to the Public Health Surveillance System. The report of all acute and chronic cases of Chagas disease began from 2008 to 2017. Currently, only acute cases and chronic cases (in children and pregnant women) of Chagas disease are mandatory notification in Colombia. This estimate is lower than the estimate from a recent systematic review in Brazil, which found a prevalence of 4.2% (95% CI: 3.1–5.7) that was calculated based on 44 population studies conducted between 1980 and 2011 in 18 Brazilian states [32].

This study shows that the Orinoco region has the highest prevalence of cases infected with *T*. *cruzi* (7.0%, 95% CI: 2.2–12.6). This result is consistent with recent entomological studies that report this region as a high endemic area due to the co-existence of domiciliary and sylvatic *T*. *cruzi* cycles [33,34]. In addition, in this region, there are reports of a natural infection index of 67% in triatomines and a high prevalence of *T*. *cruzi* infection of 89% in *Didelphis marsupialis*, the main reservoir [33].

Previous studies have reported that the Orinoco region has high infestation indexes of triatomines in palm trees, particularly *Attalea butyracea and Elaeis guineensis*, crops that are grown for economic purposes [34–36]. These palms are also used for roofing in rural dwellings, and the fruit is used to prepare juice and wine, which increases the risk of vector and oral transmission [34,36].

This study also reveals a higher prevalence of cases infected with *T*. *cruzi* in the adult population (3.0%, 95% CI: 1.0–5.0), which shows the need to rethink vector-based Chagas control strategies and to generate integrated action plans based on the patients, in order to promote self-care and patient empowerment and to reduce the burden of the disease.

Given the current migration of rural people infected with *T*. *cruzi* to urban areas, transmission through blood transfusion, organ transplantation from an infected donor or from mother to child have become important sources of new cases [37,38]. In response, several governmental initiatives have been focusing on insecticide-spraying campaigns against triatomine vectors and the compulsory screening for *T*. *cruzi* infection in blood banks [39]. In the present study, a low prevalence of Chagas disease was observed in blood donors (0.5%, 95% CI: 0.0–1.4). This shows the favorable impact that the anti-*T*. *cruzi* screening policies have had on reducing the number of blood transfusion transmitted cases, contributing to the control of the infection [40].

On the other hand, this study shows an important prevalence in pregnant women (3.0%, 95% CI: 3.0–4.0). This result contrasts with previous studies that have reported a lower prevalence of Chagas in pregnant women, such as in Brazil (1.1%, 95% CI: 0.6–2.0) [41]. Moreover, it highlights the importance of the universal screening of pregnant women for the early diagnosis and prompt treatment of infected newborns. At present, there is no national Colombian policy that establishes the control of Chagas disease in pregnant women and their newborns. In addition, the strategy of screening pregnant women to control and treat newly diagnosed infected newborns has also been shown to be cost-effective [42,43].

In general, the prevalence was lower in children. The highest prevalence of 1.0% (95% CI: 0.0–2.0) was found in the age group of over 15-year-olds. These results coincide with those of another meta-analysis conducted in Brazil, in which a higher prevalence was reported in the group with individuals older than 60 (17.7%, 95% CI: 11.4–26.5) [32]. This cohort effect was described in a study conducted in the town of Bambuí, Brazil, in which transmission of the disease was interrupted [44]. The authors reported that successful vector control campaigns reduced infections in people younger than 40 years and reported worse health indicators among seropositive subjects older than 60 years [44]. Undoubtedly, the greater survival of people with Chagas disease and the association with other chronic diseases will represent a great challenge for health systems [11,45].

In this study, there was not enough evidence to support a predominance of Chagas disease in men or women. These results contrast with those of previous studies conducted in Brazil that report a higher prevalence in women [32]. In addition, it is observed that apparently the prevalence of the disease has decreased over the years. In the context of people with chronic Chagas disease, it is important to consider the available evidence about the etiological treatment of *T*. *cruzi* infection [46–50].

The main limitations of this study include a possible publication bias because non-significant findings are less likely to be published, and that may have inflated the prevalence estimates of Chagas disease. On the other hand, most of the included studies were not representative of the national population and observed *T*. *cruzi* prevalence data are lacking in some regions. In addition, there was not enough information on the distribution of age, sex and ethnicity in each study. It was not possible to assess whether the prevalence of Chagas disease differed between ethnic groups. Potential sources of heterogeneity included regional differences, rural versus urban settings, different population groups and different age groups. Although there was considerable heterogeneity between the studies, our review provides the best and most complete estimate of the prevalence of Chagas disease in the general population and allows the comparison of the prevalence among several interest groups. The strengths of this review include its rigorous selection of studies, the objective quality assessment of the included studies and the detailed set of methods used for the analyses, including meta-regression.

In conclusion, the systematic review and meta-analysis conducted in this study indicates that the *T*. *cruzi*-infected population is aging, and that the prevalence of Chagas disease is higher in the adult population. With the aging population, the number of people with Chagas disease is likely to increase substantially over the coming decades. Based on the results of this systematic review, Chagas disease continues to be a public health problem in Colombia, with the highest prevalence of *T*. *cruzi* infection in the Orinoco region, specifically in the department of Casanare. Despite the adoption of campaigns to interrupt the main vector and screening in blood banks, the number of people who are still chronically infected will represent a high economic burden for the health system in the foreseeable future. These results highlight the need to overcome the social and economic obstacles to maintain screening and surveillance programs to identify people with chronic *T*. *cruzi* infection and thus prevent further transmission, reducing the burden of the disease and providing opportunities for secondary and tertiary prevention.

## Supporting information

S1 File PRISMA checklist. (DOCX)

S1 Table Search strategy. (DOCX)

S2 Table Studies on prevalence of Chagas disease in Colombia from 2007 to November 2017. (PDF)

S3 Table Sensitivity analyses of pooled prevalence of Chagas disease in Colombia. (DOCX)

S4 Table Univariate and multivariate meta-regression for prevalence of Chagas disease in Colombia. (DOCX)

S5 Table Risk of bias assessment of included studies using the *Hoy* 2012 tool. (DOCX)

S1 Fig Bubble plot of meta-regression results for prevalence of Chagas disease by data of data collection. (TIF)

S1 Certificate Certificate AJE. (PDF)
