# Supplementary material for: Prevalence of Chagas disease in Colombia: A systematic review and meta-analysis
Source: PLoS One. 2019 Jan 7;14(1):e0210156. doi: 10.1371/journal.pone.0210156 (PMC6322748; doi:10.1371/journal.pone.0210156)
Supplement: S1 Table — (DOCX) [file pone.0210156.s002.docx]

| **Search criteria** | **PubMed** | **Scielo** | **LILACS** | **Embase** |
| --- | --- | --- | --- | --- |
| Chagas disease AND Prevalence | 131 | 13 | 26 | 79 |
| *Trypanosoma cruzi* AND Prevalence | 1927 | 193 | 550 | 1904 |
| American trypanosomiasis AND Prevalence | 3599 | 26 | 989 | 194 |
| American trypanosomiasis AND Prevalence AND Colombia | 174 | 3 | 40 | 7 |
| *Trypanosoma cruzi* AND Prevalence AND Colombia | 137 | 14 | 32 | 67 |
| *Trypanosoma cruzi* AND Colombia | 367 | 84 | 105 | 525 |
| Chagas disease OR *Trypanosoma cruzi* AND Prevalence AND Colombia | 132 | 8 | 26 | 55 |
| Chagas disease OR *Trypanosoma cruzi* AND Epidemiology AND Colombia | 125 | 10 | 23 | 102 |
| *Trypanosoma cruzi* AND Epidemiology AND Colombia | 125 | 11 | 27 | 116 |
| American trypanosomiasis AND Epidemiology AND Colombia | 164 | 1 | 36 | 15 |
| Chagas disease OR *Trypanosoma cruzi* AND Prevalence AND Colombia | 177 | 10 | 33 | 71 |
| Chagas disease OR *Trypanosoma cruzi* AND Prevalence AND Epidemiology AND Colombia | 167 | 1 | 33 | 34 |
