# Supplementary material for: Prevalence of Chagas disease in Colombia: A systematic review and meta-analysis
Source: PLoS One. 2019 Jan 7;14(1):e0210156. doi: 10.1371/journal.pone.0210156 (PMC6322748; doi:10.1371/journal.pone.0210156)
Supplement: S2 Table — (PDF) [file pone.0210156.s003.pdf]

| Authors, publication year | Age (years) | Region | Study site | Municipality | n | Cases | Sample size | Total cases |
|---|---|---|---|---|---|---|---|---|
| Suescún-Carrero et al. 2017 | 14-48 | Andes | Boyacá | Soatá | 122 | 4 | 566 | 14 |
| | | | | Covarachia | 6 | 0 | | |
| | | | | Tipacoque | 8 | 0 | | |
| | | | | Boavita | 32 | 0 | | |
| | | | | Susacón | 2 | 0 | | |
| | | | | La Uvita | 2 | 0 | | |
| | | | | Chitaraque | 36 | 3 | | |
| | | | | San José de Pare | 44 | 0 | | |
| | | | | Moniquirá | 167 | 3 | | |
| | | | | Togüi | 49 | 1 | | |
| | | | | Santana | 95 | 3 | | |
| | | | | Zetaquira | 3 | 0 | | |
| Flórez et al. 2016 | 2-64 | Amazonas | Amazonas | Amazonas | 1130 | 1 | 1130 | 1 |
| | | | Guaviare | Guaviare | 1162 | 24 | 1162 | 24 |
| | | | Vaupés | Vaupés | 1137 | 9 | 1137 | 9 |
| Angulo-Silva et al. 2016 | 0-88 | Orinoquía | Casanare | Aguazul | 492 | 57 | 492 | 57 |
| | | | | Maní | | | | |
| Monroy et al. 2016 | 32 | Andes | Boyacá | Socotá | 138 | 2 | 138 | 2 |
| Castellanos-Domínguez et al. 2016 | 13-46 | Andes | Santander | Curiti | 30 | 1 | 1518 | 49 |
| | | | | Charalá | 14 | 0 | | |
| | | | | Coromoro | 38 | 2 | | |
| | | | | Mogotes | 115 | 21 | | |
| | | | | Onzaga | 25 | 0 | | |
| | | | | San Gil | 226 | 4 | | |
| | | | | San Joaquín | 49 | 2 | | |
| | | | | Gámbita | 13 | 0 | | |
| | | | | Guacamayo | 23 | 0 | | |
| | | | | Socorro | 329 | 5 | | |
| | | | | Suaita | 40 | 0 | | |
| | | | | Oiba | 139 | 1 | | |
| | | | | Hato | 4 | 0 | | |
| | | | | Palmas del Socorro | 3 | 0 | | |
| | | | | Simacota | 15 | 0 | | |
| | | | | Capitanejo | 95 | 4 | | |

| | | | | | | | | |
|---|---|---|---|---|---|---|---|---|
| | | | | Concepción | 25 | 0 | | |
| | | | | Enciso | 8 | 2 | | |
| | | | | Macaravita | 37 | 2 | | |
| | | | | Molagavita | 53 | 2 | | |
| | | | | Málaga | 187 | 1 | | |
| | | | | San José de Miranda | 41 | 1 | | |
| | | | | San Miguel | 9 | 1 | | |
| Bianchi et al 2015 | 4-19 | Orinoquía | Casanare | Nunchía | 1976 | 46 | 3033 | 62 |
| | | | | Yopal | 1053 | 12 | | |
| | | | | Maní | 4 | 4 | | |
| Cantillo-Barraza et al 2015 | <15 | Caribe | Bolívar | Mompós | 293 | 2 | 803 | 2 |
| | | | | Talaigua | 510 | 0 | | |
| Mejía-Jaramillo et al. 2014 | NR | Caribe | Magdalena | Sierra Nevada de Santa Marta | 214 | 79 | 214 | 79 |
| Cantillo-Barraza et al 2014 | 1-91 | Caribe | Bolívar | Mompós | 743 | 13 | 743 | 13 |
| Rocha-Muñoz et al 2014 | 18-65 | Andes | Cesar | Valledupar | 16661 | 24 | 16661 | 24 |
| Gutierrez et al 2013 | 15-89 | Orinoquía | Casanare | Hato Corozal | 71 | 8 | 486 | 75 |
| | | | | Nunchía | 61 | 19 | | |
| | | | | Paz de Ariporo | 183 | 27 | | |
| | | | | Pore | 74 | 14 | | |
| | | | | Trinidad | 97 | 7 | | |
| Manrique-Abril et al 2013 | 14-43 | Andes | Boyacá | Moniquirá | 358 | 10 | 659 | 22 |
| | | | | Miraflores | 301 | 12 | | |
| Bedoya et al 2012 | 18-65 | Andes | Antioquia | Medellín | 54499 | 4 | 54499 | 4 |
| Cucunubá et al 2012 | 13-46 | Orinoquía | Casanare | Yopal | 541 | 15 | 982 | 39 |
| | | | | Aguazul | 86 | 3 | | |
| | | | | Taurammena | 77 | 0 | | |
| | | | | Paz de Ariporo | 47 | 1 | | |
| | | | | San Luis de Palenque | 41 | 3 | | |
| | | | | Trinidad | 44 | 3 | | |
| | | | | Pore | 33 | 2 | | |
| | | | | Maní | 21 | 2 | | |
| | | | | Monterrey | 21 | 1 | | |
| | | | | Nunchía | 20 | 5 | | |
| | | | | Hato Corozal | 15 | 2 | | |

| | | | | | | | | |
|---|---|---|---|---|---|---|---|---|
| | | | | Orocué | 13 | 1 | | |
| | | | | Támara | 10 | 1 | | |
| | | | | Sácama | 3 | 0 | | |
| | | | | Recetor | 3 | 0 | | |
| | | | | Chámeza | 3 | 0 | | |
| | | | | Villanueva | 2 | 0 | | |
| | | | | Sabanalarga | 1 | 0 | | |
| | | | | La Salina | 1 | 0 | | |
| Ríos-Osorio et al 2012 | NR | Caribe | Magdalena | Sierra Nevada de Santa Marta | 355 | 119 | 355 | 119 |
| Hoyos et al 2007 | NR | Caribe | Sucre | Morroa | 122 | 1 | 122 | 1 |
| | | | | **TOTAL** | 84700 | 596 | 84700 | 596 |

NR: Not reported
