# Supplementary material for: Prevalence of Chagas disease in Colombia: A systematic review and meta-analysis
Source: PLoS One. 2019 Jan 7;14(1):e0210156. doi: 10.1371/journal.pone.0210156 (PMC6322748; doi:10.1371/journal.pone.0210156)
Supplement: S3 Table — (DOCX) [file pone.0210156.s004.docx]

| **Models** | **Available Studies for Analysis** | **Prevalence (%)**  **(95% CI)** | **Heterogeneity Test** | |
| --- | --- | --- | --- | --- |
|  |  |  | **I^2^ (%)** | **p Value** |
| 1. Exclude studies with sample size <400 | 12 | 2.0 (1.0-4.0) | 98.7 | <0.001 |
| 2. Exclude studies that did not report sex of participants | 8 | 3.0 (0.1-8.0) | 99.2 | <0.001 |
| 3. Exclude studies that year of data collection was not reported | 14 | 4.0 (2.0-6.0) | 99.1 | <0.001 |
| 4. Exclude studies that sex proportion, year of date collection and study setting was not reported | 8 | 3.0 (0.1-8.0) | 99.2 | <0.001 |
