# Supplementary material for: Prevalence of Chagas disease in Colombia: A systematic review and meta-analysis
Source: PLoS One. 2019 Jan 7;14(1):e0210156. doi: 10.1371/journal.pone.0210156 (PMC6322748; doi:10.1371/journal.pone.0210156)
Supplement: S4 Table — (DOCX) [file pone.0210156.s005.docx]

| **Covariate** | **Univariate analysis** | | | **Multivariate analysis** | | |
| --- | --- | --- | --- | --- | --- | --- |
|  | **Coefficient** | **95% CI** | ***p-*value** | **Coefficient** | **95% CI** | ***p-*value** |
| **Sample size** |  |  |  |  |  |  |
| >400 | Reference | | | | | |
| ≤400 | 1.69 | (-0.91 - 4.30) | 0.185 | 1.73 | (-1.12 - 4.58) | 0.212 |
|  |  |  |  |  |  |  |
| **Region** |  |  |  |  |  |  |
| Amazon | Reference | | | | | |
| Andean | -0.51 | (-7.23 - 6.20) | 0.853 | - | - | - |
| Caribbean | 1.33 | (-1.59 - 3.46) | 0.441 | - | - | - |
| Orinoco | 1.82 | (-1.53 - 5.19) | 0.182 | 0.33 | (-3.16 - 3.83) | 0.722 |
|  |  |  |  |  |  |  |
| **Year of publication** |  |  |  |  |  |  |
| 2007-2013 | Reference | | | - | - | - |
| 2014-2017 | 0.93 | (-0.42 - 0.61) | 0.706 | - | - | - |
|  |  |  |  |  |  |  |
| **Year data collected** |  |  |  |  |  |  |
| 2003-2010 | Reference | | | - | - | - |
| 2011-2017 | -1.31 | (-3.59 - 0.95) | 0.234 | - | - | - |
|  |  |  |  |  |  |  |
| **Participant sex report** |  |  |  |  |  |  |
| Yes | Reference | | | - | - | - |
| No | -0.31 | (-2.67 - 2.65) | 0.784 | - | - | - |
|  |  |  |  |  |  |  |
