# Supplementary material for: Prevalence of Chagas disease in Colombia: A systematic review and meta-analysis
Source: PLoS One. 2019 Jan 7;14(1):e0210156. doi: 10.1371/journal.pone.0210156 (PMC6322748; doi:10.1371/journal.pone.0210156)
Supplement: S5 Table — (DOCX) [file pone.0210156.s006.docx]

| **Study** | **Representation** | **Sampling** | **Random selection** | **Non response bias** | **Data collection** | **Case definition** | **Reliability of tool** | **Method of data collection** | **Prevalence period** | **Numerators and denominators** | **Summary assessment** |
| --- | --- | --- | --- | --- | --- | --- | --- | --- | --- | --- | --- |
| Suescún-Carrero 2017 | High risk | Low risk | Unclear risk | Low risk | Low risk | Low risk | Low risk | Low risk | Low risk | Low risk | Low |
| Flórez 2016 | Low risk | Low risk | Low risk | Low risk | Low risk | Low risk | Low risk | Low risk | Low risk | Low risk | Low |
| Angulo-Silva 2016 | High risk | High risk | High risk | Unclear risk | Unclear risk | Low risk | Unclear risk | Unclear risk | Low risk | Unclear risk | High |
| Castellanos-Domínguez 2016 | High risk | Low risk | High risk | Low risk | Low risk | Low risk | Low risk | Low risk | Low risk | Low risk | Low |
| Bianchi 2015 | High risk | Unclear risk | Unclear risk | Unclear risk | Low risk | Low risk | Low risk | Low risk | Low risk | Low risk | Medium |
| Cantillo-Barraza 2015 | High risk | High risk | High risk | High risk | Unclear risk | Low risk | Low risk | Unclear risk | Unclear risk | Unclear risk | High |
| Cantillo-Barraza 2014 | High risk | Unclear risk | High risk | Unclear risk | Unclear risk | Low risk | Low risk | Unclear risk | Unclear risk | Unclear risk | High |
| Rocha-Muñoz 2014 | High risk | Unclear risk | Unclear risk | Unclear risk | Low risk | Low risk | Low risk | Low risk | Low risk | Low risk | Medium |
| Gutierrez 2013 | High risk | Low risk | Unclear risk | Low risk | Low risk | Low risk | Low risk | Low risk | Low risk | Low risk | Low |
| Manrique-Abril 2013 | High risk | Unclear risk | Unclear risk | Unclear risk | Low risk | Low risk | Low risk | Low risk | Low risk | Low risk | Medium |
| Bedoya 2012 | High risk | Low risk | Unclear risk | Unclear risk | High risk | Low risk | Low risk | Unclear risk | Low risk | Unclear risk | High |
| Cucunubá 2012 | High risk | Low risk | High risk | Low risk | Low risk | Low risk | Low risk | Low risk | Low risk | Low risk | Low |
