# Supplementary figures and images for: Prevalence of Chagas disease in Colombia: A systematic review and meta-analysis
Source: PLoS One. 2019 Jan 7;14(1):e0210156. doi: 10.1371/journal.pone.0210156 (PMC6322748; doi:10.1371/journal.pone.0210156)

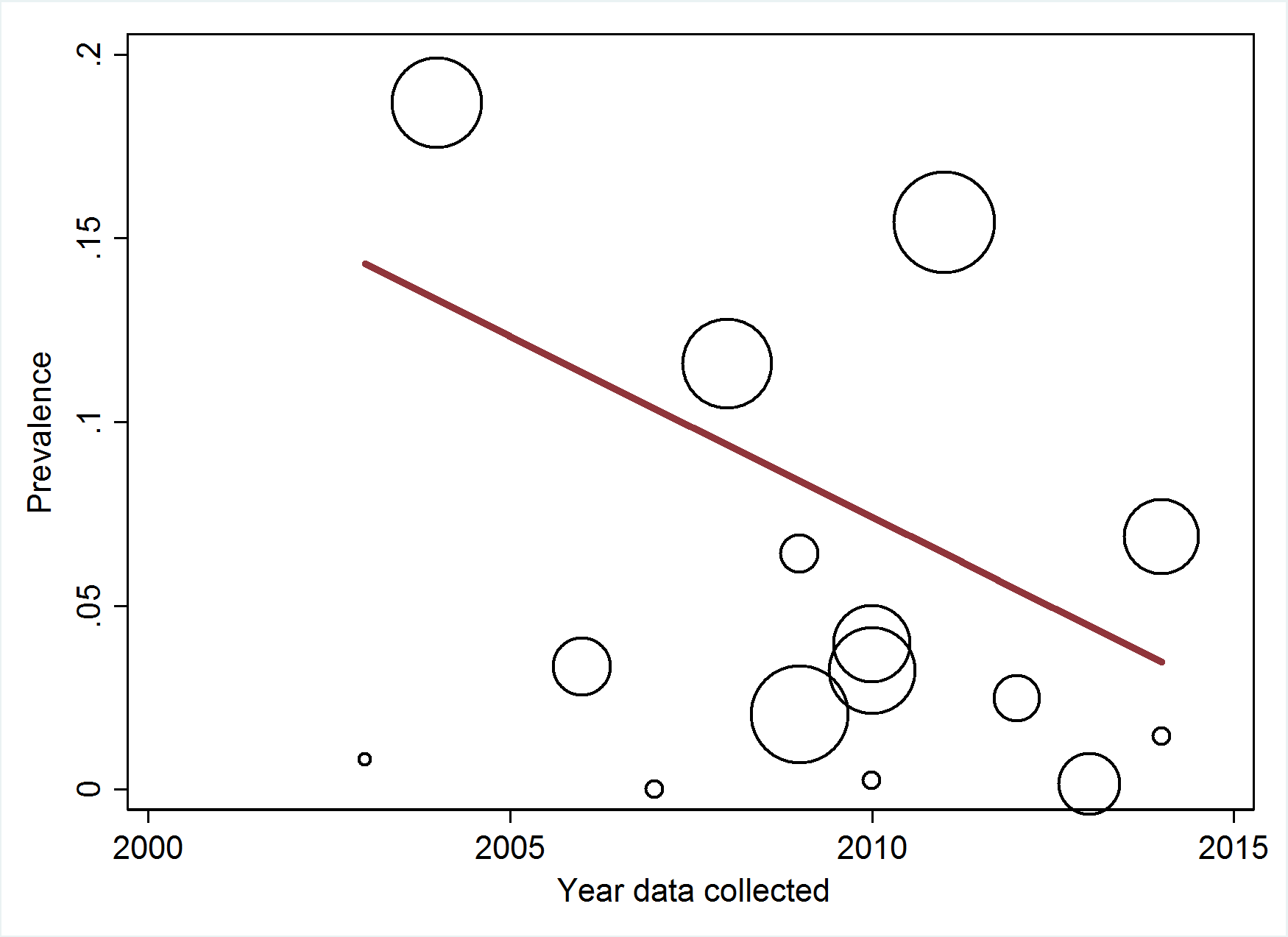

Supplement: S1 Fig — (TIF) [file pone.0210156.s007.tif]
